# A variable mineralization time and solution concentration intervene in the microstructure of biomimetic mineralized collagen and potential osteogenic microenvironment

**DOI:** 10.3389/fbioe.2023.1267912

**Published:** 2023-12-06

**Authors:** Xiujie Zhu, Haotian Bai, He Liu, Zhonghan Wang, Yao Wang, Jiaxin Zhang, Jiaqi Liu, Hui Wang, Jincheng Wang

**Affiliations:** ^1^ Department of Orthopedics, The Second Hospital of Jilin University, Changchun, China; ^2^ Orthopaedic Research Institute of Jilin Province, Changchun, China

**Keywords:** mineralized collagen, mineralization time, mineralization solution concentration, osteogenic microenvironment, microstructure, physicochemical properties

## Abstract

The absence of a conducive bone formation microenvironment between fractured ends poses a significant challenge in repairing large bone defects. A promising solution is to construct a bone formation microenvironment that mimics natural bone tissue. Biomimetic mineralized collagen possesses a chemical composition and microstructure highly similar to the natural bone matrix, making it an ideal biomimetic bone substitute material. The microstructure of biomimetic mineralized collagen is influenced by various factors, and its biomineralization and microstructure, in turn, affect its physicochemical properties and biological activity. We aimed to utilize mineralization time and solution concentration as variables and employed the polymer-induced liquid precursor strategy to fabricate mineralized collagen with diverse microstructures, to shed light on how mineralization parameters impact the material microstructure and physicochemical properties. We also investigated the influence of microstructure and physicochemical properties on cell biocompatibility and the bone-forming microenvironment. Through comprehensive characterization, we examined the physical and chemical properties of I-EMC under various mineralization conditions and assessed the *in vitro* and *in vivo* biocompatibility and osteogenic performance. By investigating the relationship between mineralization parameters, material physicochemical properties, and osteogenic performance, we revealed how microstructures influence cellular behaviors like biocompatibility and osteogenic microenvironment. Encouragingly, mineralization solutions with varying concentrations, stabilized by polyacrylic acid, successfully produced intrafibrillar and extrafibrillar mineralized collagen. Compared to non-mineralized collagen, all mineralized samples demonstrated improved bone-forming performance. Notably, samples prepared with a 1× mineralization solution exhibited relatively smooth surfaces with even mineralization. Extending the mineralization time enhanced the degree of mineralization and osteogenic performance. Conversely, samples prepared with a 2× mineralization solution had rough surfaces with large calcium phosphate particles, indicating non-uniform mineralization. Overall, our research advances the potential for commercial production of mineralized collagen protein products, characterized by dual biomimetic properties, and their application in treating various types of bone defects.

## 1 Introduction

Fractures, various pathological elements, and other factors can result in bone defects, especially larger ones, which pose a significant clinical challenge in the field of orthopedics due to the disruption of the osteogenic microenvironment between fracture ends (Li et al., 2018). Currently, effective repair methods for these bone defects include bone transport, bone grafting, and biomaterial implantation (Huang et al., 2016). The shared objective of these methods is to establish a favorable osteogenic microenvironment to facilitate bone regeneration and repair the defects. Such a conducive osteogenic microenvironment is vital for the successful treatment of bone defects. However, both autologous and allogeneic bone grafts have limitations in their clinical applications, such as limited availability of suitable donors, higher failure rates, and the risk of host rejection (Agarwal and García, 2015). Consequently, the implantation of bone biomaterials is considered a promising approach to address bone defects.

Excellent bone repair materials must create an optimal microenvironment for bone regeneration. Natural bone is a complex hierarchical composite material, consisting of organic collagen proteins and inorganic minerals. Mineral crystals are found within and between collagen fibers, and on the fiber surfaces, resulting in intrafibrillar and extrafibrillar mineralized collagen (I-EMC) (Reznikov et al., 2014a; Yu and Wei, 2021). Mineralized collagen represents the second level in the nine hierarchical levels of natural bone (Reznikov et al., 2014b), forming the nanoscale foundation responsible for the exceptional mechanical and biological properties of bone (Oosterlaken et al., 2021). Guided by progress in biomimetics and insights from *in vivo* biomineralization, researchers have become proficient at crafting various forms of *in vitro* mineralized collagen, especially extrafibrillar (EMC) and intrafibrillar mineralized collagen (IMC). These synthesized counterparts closely resemble the chemical makeup and microstructure of natural bone matrices. The differences between EMC and IMC lie in their *ex-vivo* biomimetic preparation methods and nanostructural attributes. For instance, EMC preparation largely adopts the traditional ion-mediated crystallization method (Liu S. et al., 2016). This method typically uses metastable solutions, enriched in calcium and phosphate ions or various simulated body fluids, as the reaction medium. Within this process, calcium ions accumulate, aggregate, and, post-nucleation, foster the emergence of hydroxyapatite crystals on collagen fiber surfaces. In contrast, the preparation of IMC predominantly utilizes the Polymer-Induced Liquid-Precursor (PILP) pathway (Zhu et al., 2023). This approach builds upon the conventional ion-mediated crystallization strategy by incorporating acidic polymers such as polyacrylic acid (PAA) and polyaspartic acid (PAsP). These polymers, which act as analogs to non-collagen proteins, excel in binding and isolating calcium ions, effectively delaying the onset of crystallization. This interplay results in a stable and well-hydrated amorphous precursor, preventing the amorphous calcium phosphate (ACP) from clustering and converting into hydroxyapatite before integrating with the collagen fibers (Olszta et al., 2003). Such an intricate procedure facilitates the creation of continuous apatite structures inside collagen fibers. (Jee et al., 2010; Qi et al., 2018). Additionally, the addition of polyphosphate to the PILP system, given its propensity to form electrostatic bonds with collagen fibers, refines the control over apatite’s sustained growth. This procedure culminates in the creation of hierarchical IMC, which boasts layered apatite formations reminiscent of those in natural bone. Under TEM, these formations are characterized by pronounced cross-banded patterns (Liu et al., 2011; Hu et al., 2016; Ye et al., 2016). It is this structural alignment of IMC with natural bone tissue that has amplified its acknowledgment and interest within the scientific community.

The ideal mineralized collagen bone substitute material should include both EMC and IMC, with fixed volume ratios (3:1 in bone, 2:1 in dentin). However, despite scientists around the world putting in tremendous effort, this high-level biomimetic preparation method has not yet achieved a breakthrough (Chen et al., 2023). Studies have demonstrated that, when employing the PILP method, the surface of IMC shows deposits of calcium phosphate particles, indicating the occurrence of extrafibrillar mineralization, i.e., I-EMC (Hu et al., 2016; Du et al., 2022). The preparation of I-EMC involves three essential components: Type-I collagen, minerals, and non-collagen analogs (NCP) (Oosterlaken et al., 2021). Various factors (Höhling et al., 1990; Kikuchi et al., 2004; Dhand et al., 2016; Du et al., 2018) influence these elements, including collagen diameter, orientation, crosslinking degree, phosphorylation level, and NCP molecular weight and concentration (Jee et al., 2010; Zhou et al., 2017; Qi et al., 2018). These factors can impact the *in vitro* biomimetic mineralization process, thereby influencing the degree of collagen mineralization and its microstructure. The biomineralization and microstructure of the material also play a significant role in determining its physicochemical properties and biological activity (Kim et al., 2021). Furthermore, the material’s biological activity is closely tied to its physical and chemical properties (Perrier et al., 2010; Liu et al., 2014). Consequently, carefully considering and understanding these multifaceted factors is a crucial prerequisite for preparing I-EMC that effectively emulates the structure and function of natural bone and provides an optimal osteogenic microenvironment.

In preparing mineralized collagen, both the mineralization time and the concentration of the mineralizing solution are crucial parameters. They significantly impact the microstructure of the mineralized collagen, as well as the ratio of collagen to minerals (Liu et al., 2011). Importantly, by “mineralization time,” we specifically mean the duration for which collagen fibers are exposed to the mineralizing solution. However, despite their significance, there remains considerable confusion regarding the use of these two parameters. Some studies use relatively low calcium phosphate concentrations and longer mineralization times to prepare mineralized collagen (Liu et al., 2011; Yu et al., 2020; Ye et al., 2021), while others opt for higher concentrations and significantly shortened mineralization times (Liu et al., 2011; Yu et al., 2020; Ye et al., 2021). It is worth mentioning that the latter did not provide a characterization of the nanostructure of the prepared mineralized collagen. As is well known, various physicochemical factors during the process of preparing mineralized collagen can affect its nanostructure, making it impossible to determine the specific type of mineralized collagen obtained. Notably, some studies do not specify the calcium phosphate concentrations or mineralization times (Xu et al., 2016; Zhang et al., 2018; Zhang et al., 2019; Xuan et al., 2021). The absence of standardized reporting presents challenges for cross-analyzing and comparing research findings. Furthermore, it hampers the clinical translation and progress of mineralized collagen products with a more biomimetic structure and enhanced osteogenic performance. In addition, there is a shortage of comparative studies that take into account both mineralization time and mineralization solution concentration simultaneously.

**SCHEME 1:**
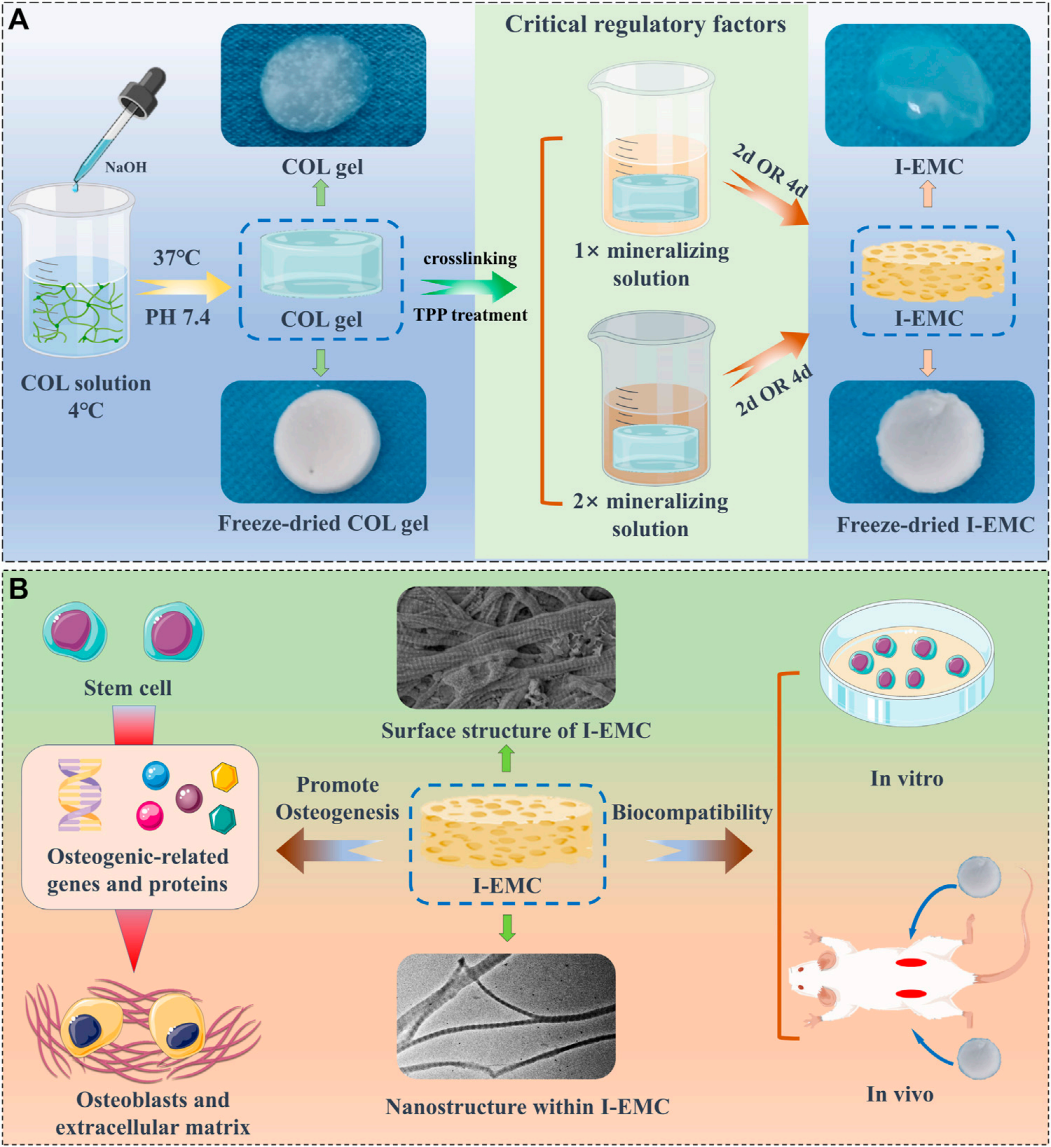
The fabrication of collagen (COL) and intra- and extra-mineralized collagen (I-EMC), exhibiting excellent biocompatibility in both *in vivo* and *in vitro* settings, along with remarkable performance in promoting osteogenic differentiation *in vitro*.

We aimed to utilize mineralization time and mineralization solution concentration as variables and adopted the PILP strategy to prepare I-EMC with distinct microstructures (Scheme 1). Through comprehensive characterization, we examined the physical and chemical properties of I-EMC under various mineralization conditions and assessed the *in vitro* and *in vivo* biocompatibility and osteogenic performance. By investigating the relationship between mineralization parameters, material physicochemical properties, and osteogenic performance, we revealed how microstructures influence cellular behaviors like biocompatibility and osteogenic microenvironment. This study has clarified the correlation between “conditions-structure-performance” in I-EMC. It establishes a foundation for the development of high-quality mineralized collagen-based bone substitute materials with improved osteogenic capabilities, and further advances their application in clinical settings. Our goal was to enhance the osteogenic microenvironment, facilitating more effective and enduring bone repair and regeneration, offering hope in the treatment of large bone defects.

## 2 Materials and methods

### 2.1 Fabrication of collagen gel

We procured a 5 mg/mL solution of Type-I collagen derived from rat tails, listed under the product number C8062, from Solarbio Life Sciences. To reconstitute the fibrils, the following steps were performed under ice bath conditions: 6 mL of Type-I collagen solution (5 mg/mL) was mixed with 3 mL of 10-fold phosphate buffered solution (PBS) buffer; deionized water was then added to adjust the collagen concentration to 1 mg/mL; after thorough mixing, 0.1 M NaOH solution was gradually added to adjust the pH to 7.4. The mixture was transferred into a 48-well culture plate with 300 μL per well and incubated at 37°C for 1 h to form a collagen gel. Subsequently, 1% glutaraldehyde solution was added for 30 min of crosslinking. After rinsing three times with deionized water, the samples were stored at 4°C for future use, and referred to as COL.

### 2.2 Preparation of I-EMC

We prepared 1 L of tris-buffered saline (TBS) buffer containing 8.77 g NaCl, 0.96 g Tris-base, and 6.61 g Tris-HCl. Before use, the buffer was filter sterilized. According to previous studies, the 1× mineralization solution is prepared as follows (Qi et al., 2018): a solution of 9 mM CaCl_2_ and 4.2 mM K_2_HPO_4_ in TBS buffer was prepared. Equal volumes of the CaCl_2_ solution and K_2_HPO_4_ solution were mixed using a syringe under magnetic stirring. Then, an appropriate amount of polyacrylic acid (PAA, MW ∼2 kDa) was added to achieve a final PAA concentration of 0.25 mg/mL. The mixture was stirred for 30 min, and the resulting mineralization solution was stored at 4°C for later use. To prepare a 2× mineralization solution, the concentrations of calcium and phosphate ions were doubled, and the PAA concentration was adjusted to 1.0 mg/mL, following the same procedure. The collagen membrane was incubated in a 2.5% sodium tripolyphosphate (TPP) solution at 37°C for 1 h, followed by washing three times with deionized water. The pre-prepared 1× and 2× mineralization solutions were separately added, and mineralization was conducted for 2 days and 4 days, respectively, with daily solution replacement. The mineralized samples were named “1X-2D,” “1X-4D,” “2X-2D,” and “2X-4D.” After mineralization, the samples were washed three times with deionized water and stored at 4°C for later use. Additionally, the conventional crystallization method was used to prepare EMC materials for comparative study.

### 2.3 Characterizations of I-EMC

#### 2.3.1 Scanning electron microscopy (SEM)

The morphologies of the collagen and I-EMC with varying microstructures were captured using a Merlin Compact SEM (Zeiss, Germany). We first processed the prepared collagen and I-EMC through freeze-drying. Subsequently, these were affixed onto the dedicated SEM sample stage using conductive adhesive and were treated via Pt sputtering before observation. Finally, the samples were placed into the SEM sample chamber and observed under an accelerating voltage of 15 kV. To identify the constituent in the samples, the energy-dispersive X-ray spectroscopy (EDS) spectra were obtained using the TEAM EDS point analysis software.

#### 2.3.2 Transmission electron microscopy (TEM)

The internal morphologies of the collagen and I-EMC with varying microstructures were analyzed using a transmission electron microscope (Tecnai F20) operated at 200 kV. To prepare the samples for TEM, we initially immersed the previously prepared collagen and I-EMC specimens into deionized water, followed by homogenization to reduce the specimens into fibrous form. We then dropped 100 μL of the suspension onto the copper grid and allowed it to air dry overnight. Notably, we conducted the observations directly without any staining process on the samples. To prepare mineralization solution TEM samples, different concentration solutions were dropped onto the copper grid.

#### 2.3.3 X-ray diffractometry (XRD)

XRD analysis of the collagen and I-EMC was meticulously conducted using a Rigaku Ultima IV X-ray diffractometer, outfitted with a copper target. Operating the diffractometer in a continuous scanning mode, with a precision step size of 0.02° across a testing range of 10°–60°, allowed for the procurement of comprehensive and uniform data.

#### 2.3.4 Fourier transform infrared spectra (FTIR)

Characterization of the surface functional groups was achieved via FTIR, employing an attenuated total reflectance (ATR) mode. The infrared spectra (ATR-FTIR) were meticulously collected over the range of 500–4,000 cm^−1^ through a sequence of 32 scans, each with a resolution of 4 cm^−1^. This process was facilitated using a Nicolet 6700 FTIR, specifically equipped with a flat-plate ATR from Thermo Fisher Scientific.

### 2.4 Water contact angle (WCA) measurements

The dynamic water contact angles of collagen and I-EMC were measured at 25°C using a dynamic contact angle analyzer (Theta Flex, Biolin Scientific). Building upon the methodologies employed by previous researchers (Ye et al., 2021), we first freeze-dried the collagen and I-EMC samples and then placed them between two glass slides to ensure their flatness. Next, the samples were carefully attached to glass slides. For the measurement process, we utilized 4 μL of distilled water. The WCAs were determined using a goniometer and the sessile drop method. Considering the superior hydrophilicity of the collagen material, we analyzed the dynamic WCA of different materials by capturing photographs at intervals of 0.03 s. Additionally, we performed measurements on three samples within the same group.

### 2.5 Thermogravimetric analysis (TGA)

The tested sample weighed between 3 and 5 mg, and a TGA was conducted utilizing a thermogravimetric analyzer (TGA2, Mettler Toledo, Switzerland). Considering the degradation temperatures of collagen and hydroxyapatite (HA), the TGA testing conditions were set as follows: the heating temperature ranged from 37°C to 800°C, with a heating rate of 10°C per minute. The TGA analysis was performed under air.

### 2.6 Isolation and culture of bone marrow stromal cells (BMSCs)

BMSCs were obtained from a healthy one-week-old female New Zealand rabbit and cultured in Dulbecco’s modified eagle medium/nutrient mixture F-12 (DMEM/F12), enriched with 10% fetal bovine serum and 1% streptomycin-penicillin. The culture medium was replaced every 3 days, and the cell cultures were nurtured in a humidified incubator set to 37°C and 5% CO_2_. Once the medium reached approximately 80% confluence, the cells were treated with trypsin at 37°C for 2 min prior to passaging. After three passages, the cells were conditioned for subsequent *in vitro* biocompatibility and osteogenesis experiments.

### 2.7 Cell viability, proliferation, and morphology

The fabricated COL and I-EMC were transferred into a new 48-well plate and subjected to sterilization with 75% ethanol. Over the next 3 h, the ethanol was refreshed hourly, followed by sterilization overnight. Before the process of cell seeding, all samples were ventilated and exposed to ultraviolet (UV) sterilization for 2 h. Subsequently, a rinse with PBS was performed to remove any potential residual solvents. The samples were then immersed in a complete culture medium for an additional 2 h. In the final step, taking cues from prior research, we seeded the third-generation BMSCs onto the membranes at a density of 5 × 10^4^ cells per well (Wang et al., 2019). We renewed the culture medium bi-daily. After 24 h, the collagen and mineralized collagen membranes, with the attached BMSCs, were relocated to a new 48-well plate. Using the calcein-AM/PI double staining kit, we assessed the vitality of the BMSCs. For the CCK-8 assay, we seeded BMSCs at a density of 2 × 10^4^ cells per well in a 48-well culture plate. One day later, we transferred the films to a new plate and evaluated cell proliferation using the CCK-8 assay (CCK8, Bioss, China) on days 1, 3, and 7 of culture. Additionally, we employed phalloidin staining to assess the morphology of cells on the membranes. The F-actin filaments and nuclei of the BMSCs were marked, respectively, using rhodamine-phalloidin and 4′,6-diamidino-2-phenylindole (DAPI). Specifically, after a 24-h incubation period, we relocated the membranes to a fresh 48-well plate, and then applied 4.0% paraformaldehyde to fix the samples. Next, we performed permeabilization using 0.5% Triton X-100. Subsequently, the samples were incubated with rhodamine-phalloidin for 30 min, followed by a 2-min counterstain with DAPI. All stained samples were captured using a fluorescence microscope (RVL-100-M, ECHO). The immunofluorescent intensity was quantified using ImageJ.

### 2.8 *In vivo* biocompatibility

Drawing upon the methods of prior researchers (Zandi et al., 2021), we conducted an *in vivo* biocompatibility study utilizing 11 male Sprague Dawley (SD) rats, each weighing approximately 200 g. Initially, we created two 1-cm incisions on the dorsal skin of each rat and prepared small subcutaneous pockets under sterile conditions. We then sequentially implanted COL, 1X-2D, 1X-4D, 2X-2D, and 2X-4D samples (four samples per group) into the subcutaneous pockets on the rat’s back, implanting two identical samples in each rat. One rat served as the control group and did not receive any sample; its incisions were directly sutured. After 7 days of sample implantation, we euthanized the rats and collected the implants and the surrounding tissues, heart, liver, spleen, lung, and kidney tissues. We then conducted hematoxylin and eosin (HE) staining and histological analysis on these tissues to evaluate the extent of inflammation.

### 2.9 Alkaline phosphatase (ALP) activity

We seeded 1 × 10^5^ BMSCs onto collagen or mineralized collagen membranes in a 48-well plate. After 1 day, we carefully transferred the membranes to a new well plate. Following prior research protocols (Zhao et al., 2020), we added osteogenic culture medium containing 10 mM of β-glycerophosphate, 50 μM of ascorbic acid-2-phosphate, and 0.1 μM of dexamethasone, with media changes every other day. After completing the 7-day osteogenic induction, ALP activity was evaluated using ALP staining and ALP assay kits.

### 2.10 Alizarin red S (ARS) staining

The procedures of cell seeding, medium replacement, and incubation followed the same steps as the ALP staining. After 14 days of osteogenic induction, the samples were fixed using 4% paraformaldehyde, washed with deionized water, and then stained for 10 min with 2% alizarin red S solution at pH 4.2. The samples were then repeatedly washed with deionized water to remove any residual alizarin red S. Finally, we observed the calcium deposits under a microscope (RVL-100-M, ECHO). Calcium phosphate in the I-EMC samples can interfere with ARS staining. Therefore, we cultured BMSCs in a blank plate and co-cultured them with the different samples. After 14 days of osteogenic induction, the cells at the bottom of the plate were stained with alizarin red. After thoroughly removing unbound dyes with deionized water, we captured images using a microscope (RVL-100-M, ECHO). The Alizarin Red-stained calcium nodules were then eluted from the cultures using the cetylpyridinium chloride method (Xiao et al., 2020)), and their absorbance at 540 nm was assessed for quantification.

### 2.11 Immunocytochemistry

After 14 days of osteogenic induction, immunocytochemical staining was performed to detect the expression of osteocalcin (OCN) and runt-related transcription factor 2 (RUNX-2). The samples were fixed with 10% paraformaldehyde at room temperature for 10 min, followed by cell permeabilization with 0.1% Triton X-100 at room temperature for 20 min. To block nonspecific binding, cells were incubated with 10% goat serum at room temperature for 30 min. Subsequently, primary antibodies against OCN (A14636, AB clonal) and RUNX2 (A2851, AB clonal) diluted at 1:180 were added and incubated overnight at 4°C. Secondary antibodies (AS053, AB clonal) diluted at 1:400 were then added and incubated for 1 h at 37°C. Subsequently, phalloidin and DAPI staining were performed as described. Throughout the entire experiment, the samples were rinsed with PBS-Tween between every step. Finally, the images were observed and captured using a fluorescence microscope (RVL-100-M, ECHO), and the fluorescence intensity of each bone-related protein was semi-quantitatively analyzed using ImageJ software.

### 2.12 Statistical analysis

All data are presented as mean ± standard deviation. For data comparison, we employed one-way analysis of variance, paired *t*-test, and the Bonferroni–Dunn *post hoc* test. A significance level of *p* < 0.05 was considered statistically significant. All statistical analyses were performed using GraphPad Prism 9 (GraphPad Software). Each experiment was independently repeated at least three times.

## 3 Results and discussion

### 3.1 Microstructure and element distribution of I-EMC

**FIGURE 1:**
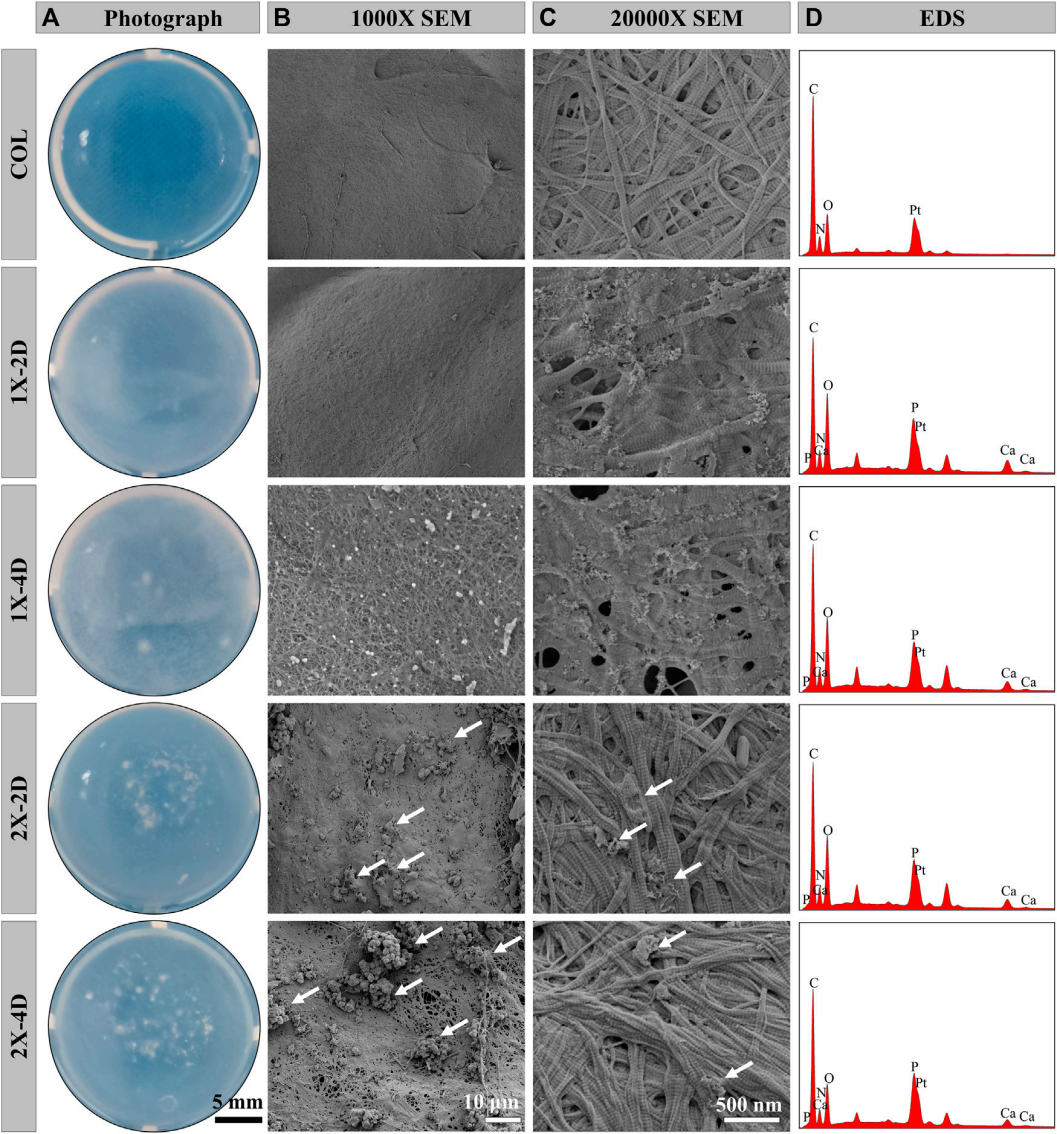
Characterizations of COL and I-EMC. Gross appearance **(A)** and SEM morphologies **(B, C)** and EDS analysis **(D)** of various samples. White arrows highlight the presence of large calcium phosphate particles.

**FIGURE 2:**
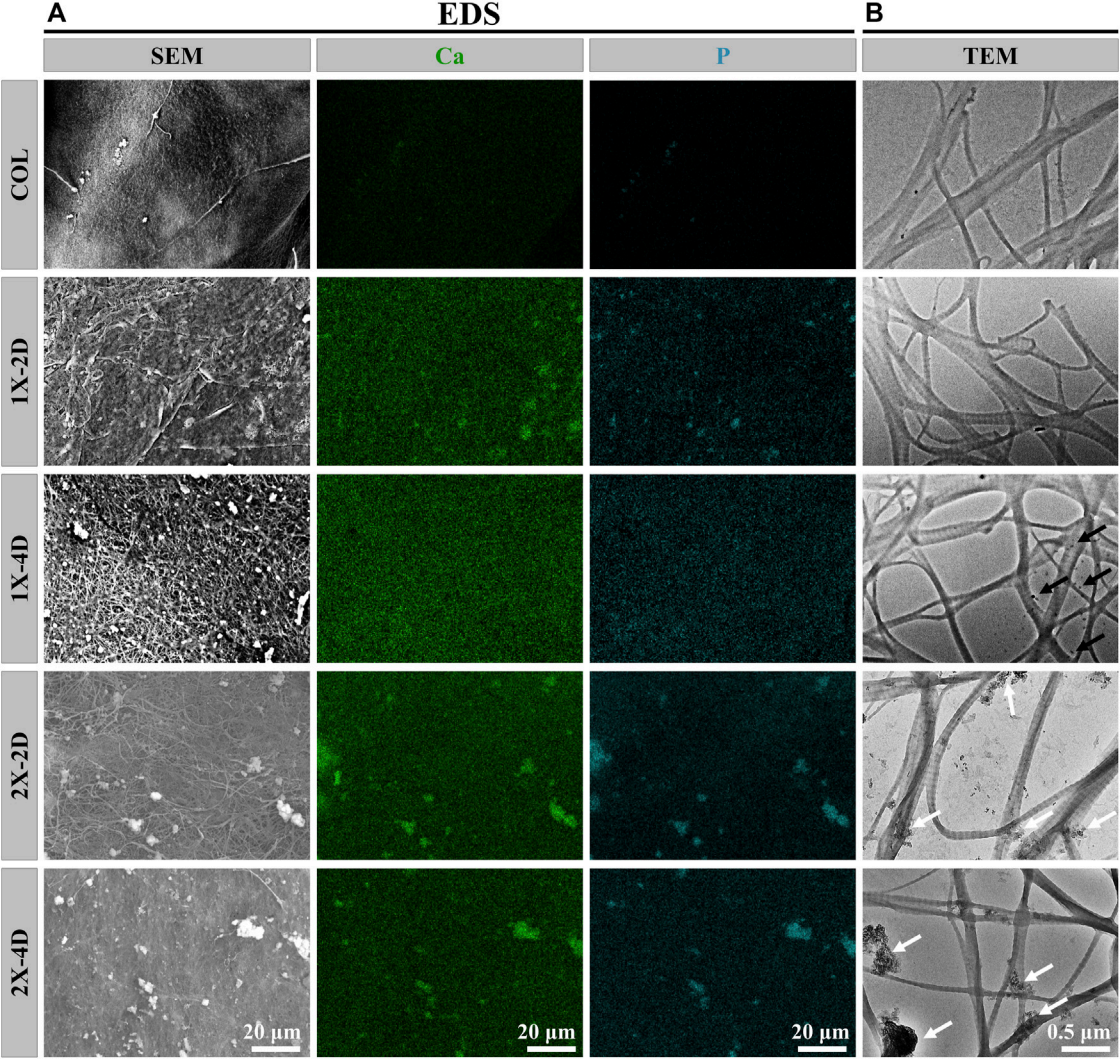
Characterization of COL and I-EMC. EDS mapping images **(A)** and TEM images **(B)** of different samples. White arrows indicate the presence of large calcium phosphate particles, while black arrows indicate the presence of small amorphous calcium phosphate nanoparticles.

We employed the PILP process to produce I-EMC with diverse microstructures. A collagen protein solution with a concentration of 1 mg/mL was prepared under 4°C conditions. After adjusting the pH, the solution was incubated at 37°C for 1 h, resulting in self-assembly of collagen proteins and the formation of collagen gel. Figure 1A shows the appearance of the collagen gel and different I-EMC samples. The collagen gel is initially transparent, but after mineralization, it transforms into a milky white, opaque appearance. Specifically, the samples mineralized with 1× mineralization solution exhibit a uniform milky white appearance, whereas the samples mineralized with 2× mineralization solution have more visible white particles scattered on the surface. The number of white particles increases with longer mineralization time. After freeze-drying, both collagen and I-EMC exhibit a white sponge-like structure, with no apparent difference in appearance between pre-mineralized and post-mineralized states (Scheme 1). Additionally, it should be noted that in the experiment, we observed that, after standard cleaning (using deionized water), some of the calcium phosphate particles on the surface of mineralized collagen were partially removed. However, during electron microscope observations (Figure 1B; Figure 2A), some residual calcium phosphate particles that were not completely eliminated from the surface were still visible. We speculate that these particles may have a relatively tight association with collagen. Although increasing the number of cleaning cycles or enhancing cleaning intensity (such as introducing ultrasonic cleaning techniques) might remove some of these particles, it is important to note that this could potentially lead to structural damage to the mineralized collagen.

Under SEM, the surface morphology of the samples is clearly revealed in Figures 1B,C. Prior to mineralization, the COL sample exhibits a smooth surface, while after mineralization, the surface of collagen protein becomes rough. Among them, the 1X-2D and 1X-4D samples show dispersed small particles on the surface with uniform features, whereas the 2X-2D and 2X-4D samples exhibit dispersed larger calcium phosphate particles with uneven distribution. At high magnification, both COL and I-EMC are composed of reconstituted collagen fiber networks, and the process of biomineralization does not interfere with their fiber structure (Figure 1C). Compared to the IMC prepared without template analog TPP (Ye et al., 2021), the introduction of TPP allowed us to clearly observe the periodic band pattern in I-EMC (Jin et al., 2019). The EDS spectrum is shown in Figure 1D. The presence of calcium and phosphorus elements indicates the deposition of calcium phosphate on the collagen protein, demonstrating the successful preparation of mineralized collagen material.

To assess the uniformity of I-EMC sample mineralization, we conducted EDS to qualitatively map the distribution of calcium and phosphorus. The results depicted in Figure 2A demonstrate that the distribution of calcium and phosphorus elements is uniform in the 1X-2D and 1X-4D samples. However, in the 2X-2D and 2X-4D samples, calcium and phosphorus elements tend to cluster and exhibit lower density in specific areas, indicating poor uniformity. These findings are consistent with the observations and SEM results shown in Figure 1. It can be inferred that an increase in the concentration of the mineralization solution leads to uneven mineralization, resulting in a higher deposition of minerals outside the fibers.

On the other hand, the collagen fibers obtained from the five different samples exhibit distinct periodic patterns, as shown in Figure 2B, indicating successful mineral deposition within the interstitial regions of the collagen fibers, resembling the D-band pattern observed in natural bone (Jin et al., 2019). This D-band pattern observed through TEM aligns with the periodic banding pattern observed through SEM, which may be the result of intermittent deposition of nano-sized calcium phosphate within the fibers upon the introduction of the template analog TPP.

**FIGURE 3:**
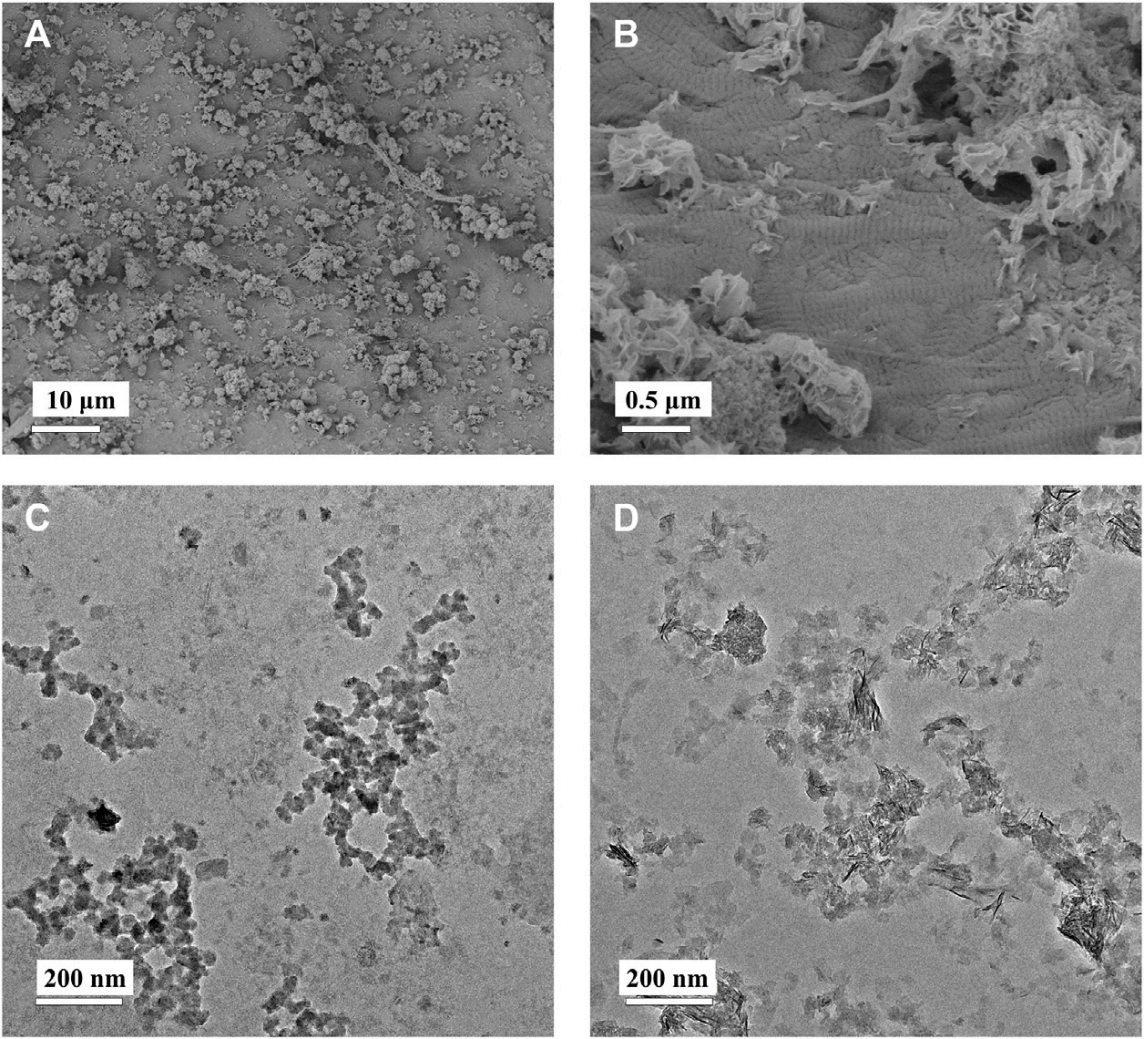
Low-magnification **(A)** and high-magnification **(B)** SEM images of EMC. PAA-stabilized 1× mineralization solution **(C)** and 2× mineralization solution **(D)** depicting TEM images of ACP.

Moreover, the 1X-4D sample reveals the presence of nano-sized amorphous calcium phosphate (ACP) particles stabilized by PAA on the surface, while the 2X-2D and 2X-4D samples exhibit larger clusters of calcium phosphate deposited on the collagen surface, resembling the surface structure of EMC (Figures 3A,B). These findings demonstrate that mineralization within collagen fibers can be achieved with different concentrations of PAA-stabilized mineralizing solutions. However, this process also leads to varying degrees of mineralization occurring outside the fibers. With an increase in mineralizing solution concentration, the stabilizing effect of PAA decreased, leading to the formation of larger-sized clusters of calcium phosphate (Figures 3C,D). As these clusters could not penetrate the interior of the collagen fibers, more substantial deposits of calcium phosphate occurred on the outer surface of the fibers. It can be inferred that, due to the limited surface area of collagen fibers, the deposition of minerals outside the fibers has an impact on the progression of internal mineralization.

### 3.2 Chemical composition and organic and inorganic content of I-EMC

**FIGURE 4:**
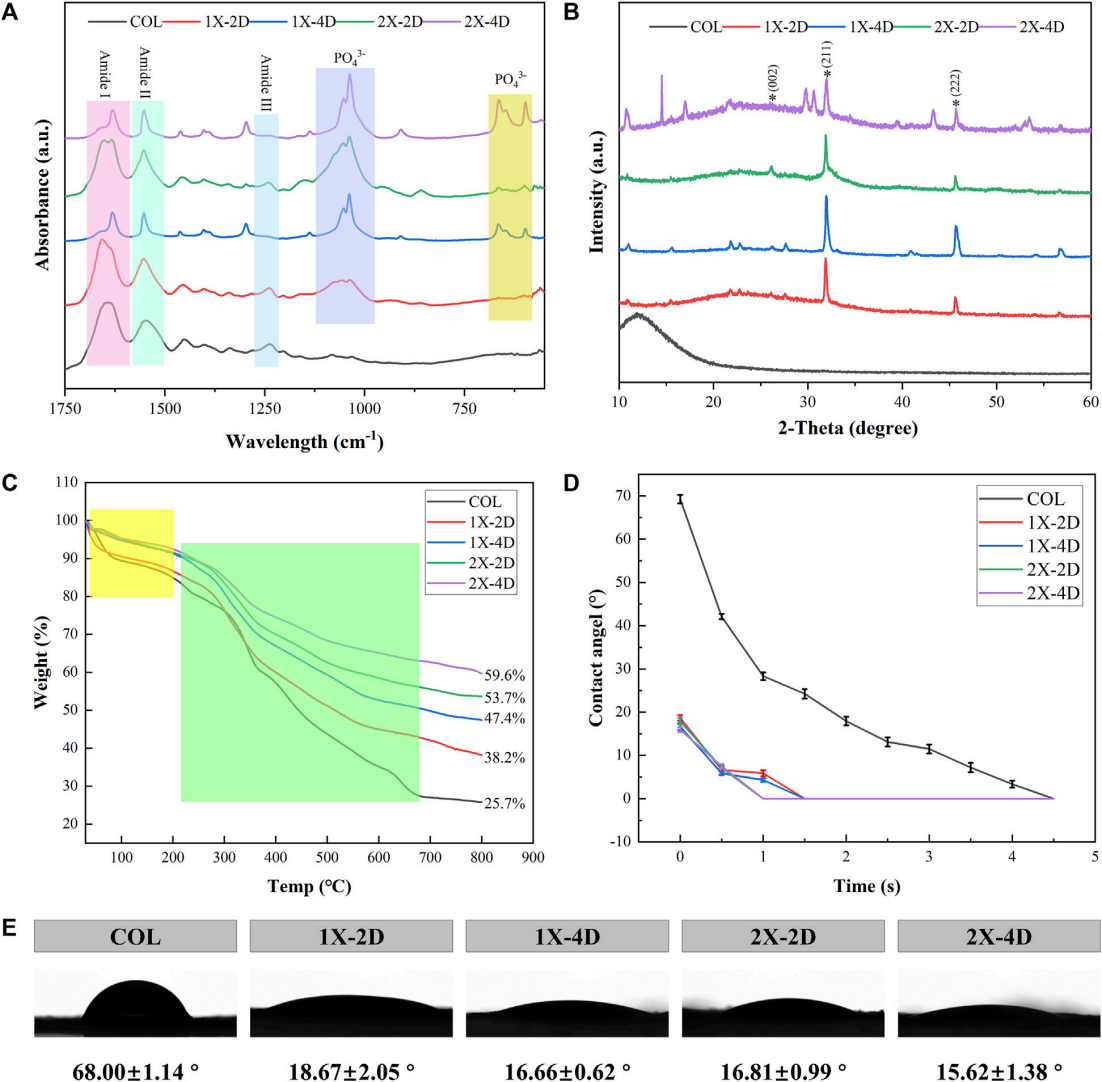
Characterization of COL and I-EMC. **(A)** FTIR spectra, **(B)** XRD spectra, **(C)** TGA curves, and **(D)** water contact angle (WCA) graph. **(E)** WCA images and average WCA.

FTIR spectroscopy provides direct information about the conformational structure of compounds through the absorption peaks of characteristic functional groups. The infrared spectra of the five sample groups are shown in Figure 4A. In the FTIR spectra, COL exhibits typical amide bands, with the absorption peak at 1,650 cm^-1^ primarily attributed to the stretching vibration of the C=O bond (amide I band), the peak at 1,550 cm^-1^ mainly arising from the vibrations of the C-N and N-H bonds (amide II band), and the peak at 1,240 cm^-1^ mainly resulting from the stretching vibrations of the C-N and C-C bonds (amide III band). The different I-EMC samples exhibit similar infrared spectral patterns. Firstly, the absorption peaks corresponding to the triple helical structure of collagen are present, indicating that the triple helical structure of collagen is well-preserved after mineralization (Du et al., 2022). In the biomimetic mineralization process, calcium phosphate ions first aggregate to form ACP, which then deposits within the collagen fibers and eventually transforms into poorly crystalline hydroxyapatite and hydroxyapatite crystals. Therefore, in addition to the aforementioned collagen protein peaks, the I-EMC samples exhibit a strong absorption peak around 1,040 cm^-1^, corresponding to the stretching vibration of phosphate groups (PO₄³⁻), as well as absorption peaks around 595 cm^-1^ and 660 cm^-1^, with the former corresponding to the vibrational absorption of phosphate groups (PO₄³⁻) in ACP and the latter corresponding to the stretching vibration absorption of hydroxyl groups (OH) in hydroxyapatite. Thus, the ACP gradually converts into hydroxyapatite during the mineralization process (Du et al., 2022). Previous studies have reported similar results (Xia et al., 2013; Hu et al., 2016; Wang et al., 2019). The sharper the peak shape, the higher the crystallinity. By observing the changes in the absorption peaks of the phosphate groups in mineralized collagen under different conditions, we found that the characteristic peaks at 595 cm^-1^, 660 cm^-1^, and 1,040 cm^-1^ were highest and sharpest in the 1X-4D and 2X-4D samples, indicating a higher content and purity of the phosphate groups. This also implies that extending the mineralization time can promote the transformation of hydroxyapatite crystals. Additionally, compared to the 1X-2D sample, the 2X-2D samples exhibited relatively higher and sharper absorption peaks, indicating that increasing the mineralization solution concentration can also enhance the content and purity of the phosphate groups and promote the transformation of hydroxyapatite crystals. However, it should be noted that, as mentioned earlier, this is due to the decreased stabilizing effect of PAA on high-concentration mineralization solution, which accelerates the transformation of ACP to hydroxyapatite crystals, resulting in more mineralization occurring outside the fibers.

The XRD spectra of the sample groups are presented in Figure 4B. COL exhibits a broad characteristic diffraction peak, known as the “D peak,” around 13°, which reflects the fibrous structure and hierarchical features of collagen. Different I-EMC samples display similar XRD spectra. The peak at 26.2° (2θ) corresponds to the (002) plane of HA. The main peak in the range of 2θ = 31.5°–33.5° corresponds to the overlapping of the (112) (211), and (300) crystal planes of apatite. This is similar to the XRD spectrum of natural cancellous bone (Li et al., 2012). The peak at 45.4° (2θ) corresponds to the (222) plane of HA. All diffraction peaks are sharp, indicating a high degree of crystallinity in the mineralized collagen. Furthermore, compared to samples mineralized for 2 days, the mineralized collagen after 4 days of mineralization exhibits relatively stronger diffraction peaks, which typically indicate higher crystallinity, consistent with the results of FTIR.

Thermogravimetric analysis (TGA) is a valuable technique used to investigate the composition, components, and stability of materials by monitoring their mass changes as the temperature increases. Figure 4C displays the TGA curves for the five sample groups, which exhibit two distinct weight loss stages. The initial weight loss, occurring within the range of 37°C–200°C, corresponds to the removal of bound water (highlighted in yellow box). Subsequently, between 200°C and 660°C, the weight loss corresponds to the degradation of collagen protein (highlighted in green box). Due to the fact that HA primarily loses weight through the release of bound water, the mass loss is minimal, with the majority occurring during the collagen degradation process. Notably, the I-EMC samples exhibit lower mass loss compared to the COL sample. A reduced weight loss in mineralized collagen suggests a higher remaining mass, indicating an elevated concentration of HA within the collagen matrix. Figure 3C depicts the mass residue rates of samples from each group at the conclusion of thermal gravimetric analysis. Based on this data, we can easily calculate the mass loss rates within the specified temperature range for each group of samples, namely, COL (74.3%), 1X-2D (61.8%), 1X-4D (52.6%), 2X-2D (46.3%), and 2X-4D (40.4%). This suggests that prolonging the mineralization time and increasing the concentration of the mineralization solution can enhance the mineral content, although higher solution concentration often leads to more mineralization occurring outside the fibers. Furthermore, it is noteworthy that the second weight loss stage of the COL sample occurs earlier than that of the I-EMC samples. This indicates that mineralized collagen exhibits higher thermal stability than pure collagen, which aligns with previous research findings ^29^. The observed changes in decomposition temperature reflect a close structural relationship between collagen and HA, suggesting that most of the crystals are embedded within the collagen fibers (Li et al., 2012; Liu et al., 2014; Qi et al., 2018; Thrivikraman et al., 2019; Ye et al., 2021).

### 3.3 Hydrophilicity of I-EMC

Collagen protein, as a common biomaterial, exhibits excellent hydrophilicity. In this study, we utilized a compression molding method to prepare the samples and conducted WCA measurements. Based on the dynamic changes in WCA over time, we plotted the dynamic contact angle curves (Figure 4D) for all samples. Figure 4E shows representative images of water droplets on the test samples, along with the corresponding average WCA and standard deviations. Through the observation of dynamic contact angle curves, we noticed a gradual decrease in WCAs during the measurement process, attributed to droplet absorption and spreading. The WCA for pure collagen was maintained for 4.5 s, while after mineralization, the contact angle was reduced to 1.5 s, indicating that mineralization increased the collagen hydrophilicity. Importantly, after 5 s, all samples exhibited superhydrophilicity, consistent with previous findings (Ye et al., 2021; Du et al., 2022). However, some researchers suggest that the attachment of HA or ACP particles on collagen surfaces may cover hydrophilic groups and, thus, reduce the material’s hydrophilicity (Du et al., 2022). We acknowledge that this could be related to the handling of samples during water contact angle testing. Overall, whether mineralized or not, all samples exhibited excellent hydrophilicity.

### 3.4 *In vitro* biocompatibility of I-EMC

**FIGURE 5:**
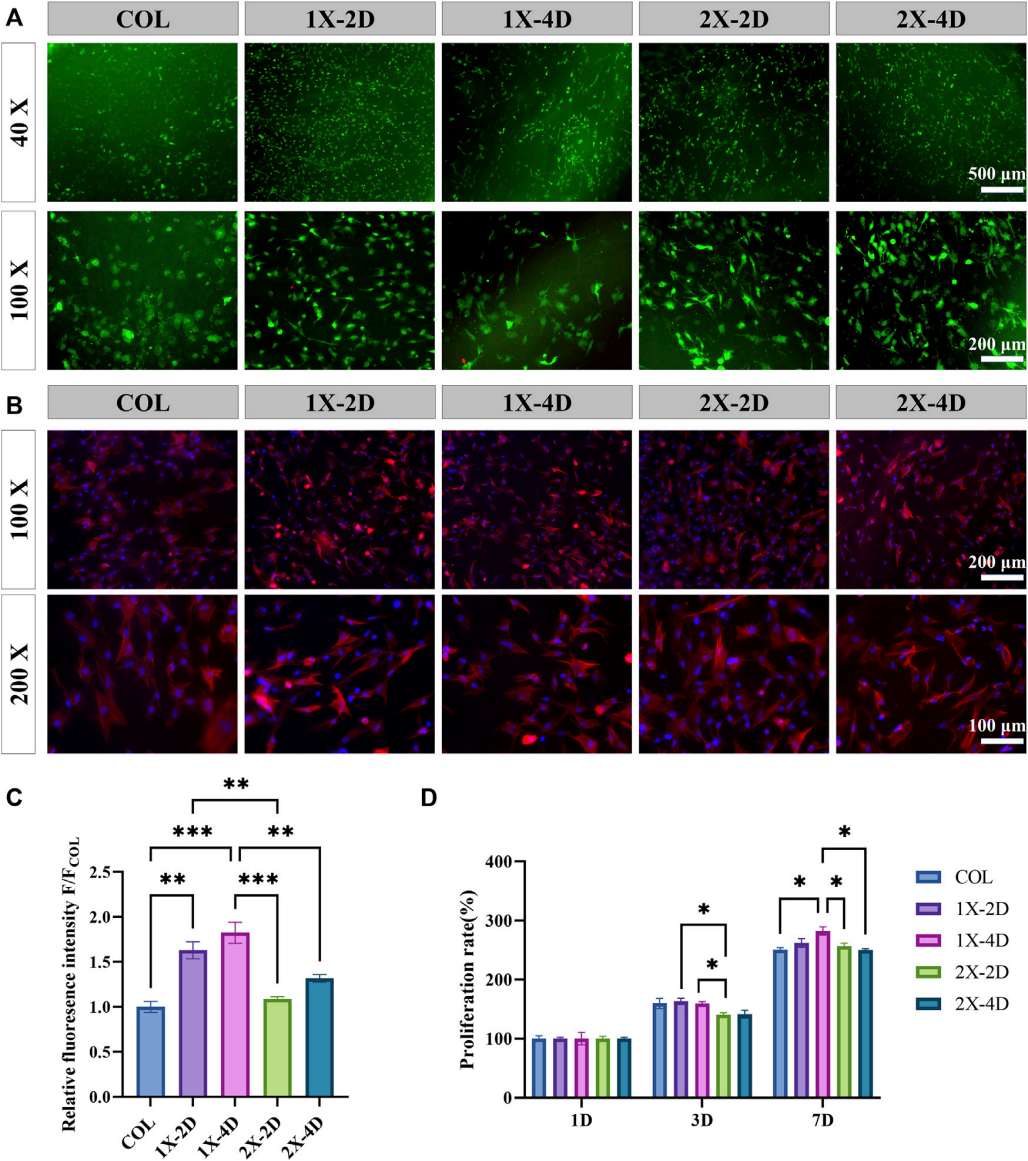
*In vitro* biocompatibility of COL and I-EMC. **(A)** Calcein-AM/PI staining of BMSCs at 24 h: live cells (green) and dead cells (red). **(B)** Fluorescent imaging with rhodamine-DAPI staining. **(C)** Quantitative analysis of F-actin fluorescence intensity (n = 3). **(D)** Cell proliferation within the different samples at 1, 3, and 7 days. (*p < 0.05, **p < 0.01, ***p < 0.001).

The hydrophilicity, morphology, and roughness of materials play a pivotal role in influencing cell adhesion, migration, and proliferation. The biocompatibility of materials serves as the fundamental basis for their biological applications. To evaluate the biocompatibility of collagen and I-EMC samples, we employed calcein-AM/PI staining to analyze the cytotoxicity of the cells. The results demonstrated that BMSCs adhered well to the surface of each sample and exhibited high cell viability after 24 h (Figure 5A), indicating good biocompatibility. Subsequently, we performed CCK-8 assays to analyze the effects of various samples on cell proliferation. BMSCs were seeded on samples, and cell proliferation was assessed after 1, 3, and 7 days after seeding. Figure 5D shows that the cell numbers in different samples significantly increased with prolonged culture time. Compared to the 2X-2D group, the 1X-2D and 1X-4D samples promoted cell proliferation on the third day, indicating better biocompatibility toward BMSCs. Furthermore, on the seventh day, the 1X-4D group significantly enhanced cell proliferation.

To observe the morphology of BMSCs adhering to the surfaces of different samples, we used rhodamine-phalloidin staining to label F-actin (Figure 5B). The results showed that, after 24 h of culture, most cells adhered to the samples and exhibited a spread-out state. Cells cultured on the COL sample had a more rounded morphology, consistent with the results observed in the live/dead staining, which may be attributed to the porous and loose structure of collagen, allowing BMSCs to easily penetrate into the collagen gel. In contrast, cells cultured on the four types of I-EMC samples exhibited an elongated morphology, indicating cell migration characteristics. We quantitatively analyzed the fluorescence intensity of F-actin using ImageJ software and presented the results in a bar graph (Figure 5C). The results showed that the F-actin fluorescence intensity was significantly higher in the 1X-2D and 1X-4D samples compared to the other groups, with no statistical difference between the two groups.

F-actin is a multifunctional protein and an important component of the cell cytoskeleton. It can form microfilaments, rapidly assemble and disassemble, providing mechanical support for cell morphology and movement (Pollard and Cooper, 2009; Cerutti and Ridley, 2017), and participate in key cellular processes such as cell migration and division (dos Remedios et al., 2003). Additionally, F-actin plays a crucial role in the osteogenic process of BMSCs (Chen et al., 2016), and the enhancement of its fluorescence intensity is beneficial for the early osteogenic differentiation of BMSCs (Wu et al., 2020). Therefore, the 1X-2D and 1X-4D samples may significantly enhance the expression of F-actin and the reorganization of the cell cytoskeleton, thereby inducing the migration and osteogenic differentiation of BMSCs. This may be attributed to the uniform mineralization and surface structure of these samples.

### 3.5 *In vivo* biocompatibility and toxicity of I-EMC

**FIGURE 6:**
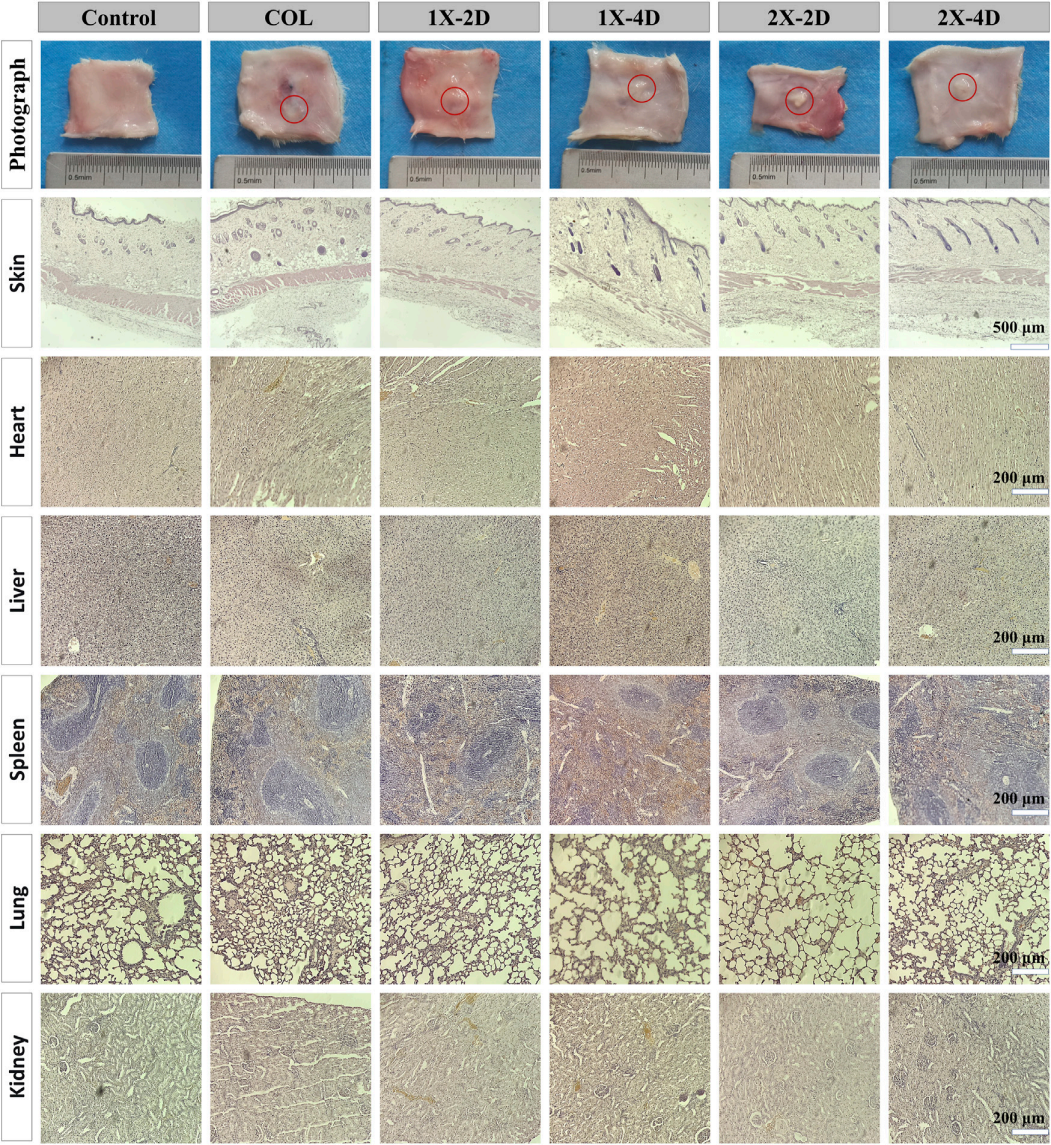
*In vivo* biocompatibility assessment of COL and I-EMC after subcutaneous implantation in rats. Macroscopic view and HE staining of the skin around the implant, as well as heart, liver, spleen, lung, and kidney tissues after 7 days of implantation. The control group comprised rats without any implanted materials. The red circle marks the location where COL and I-EMC were implanted.

Our findings indicate that both COL and I-EMC materials exhibited no cytotoxicity *in vitro* and demonstrated excellent biocompatibility. However, it should be noted that *in vitro* results may not always reflect *in vivo* performance. To further evaluate the biocompatibility of COL and I-EMC materials, an *in vivo* study was conducted using a rat subcutaneous pouch model. During the 7-day period after implantation of various samples into the subcutaneous tissue of rats, no deaths or infections were observed. Additionally, all incisions in each group healed well. Figure 6 shows the macroscopic view and HE staining of the skin around the implant materials, heart, liver, spleen, lungs, and kidneys in each group. Macroscopic observation of the samples revealed no signs of redness or local exudation. HE staining of the skin confirmed the absence of any inflammatory reactions. Additionally, to assess the material toxicity to the organism, we collected samples from the rat’s heart, liver, spleen, lungs, and kidneys, conducted sectioning, and performed HE staining. Compared to the control group, the rat’s various organ tissues exhibited intact structures without any pathological changes. In conclusion, both collagen and I-EMC materials demonstrated excellent biocompatibility, both *in vivo* and *in vitro*, supporting cell adhesion and proliferation.

### 3.6 Effect on osteogenic differentiation of BMSCs

**FIGURE 7:**
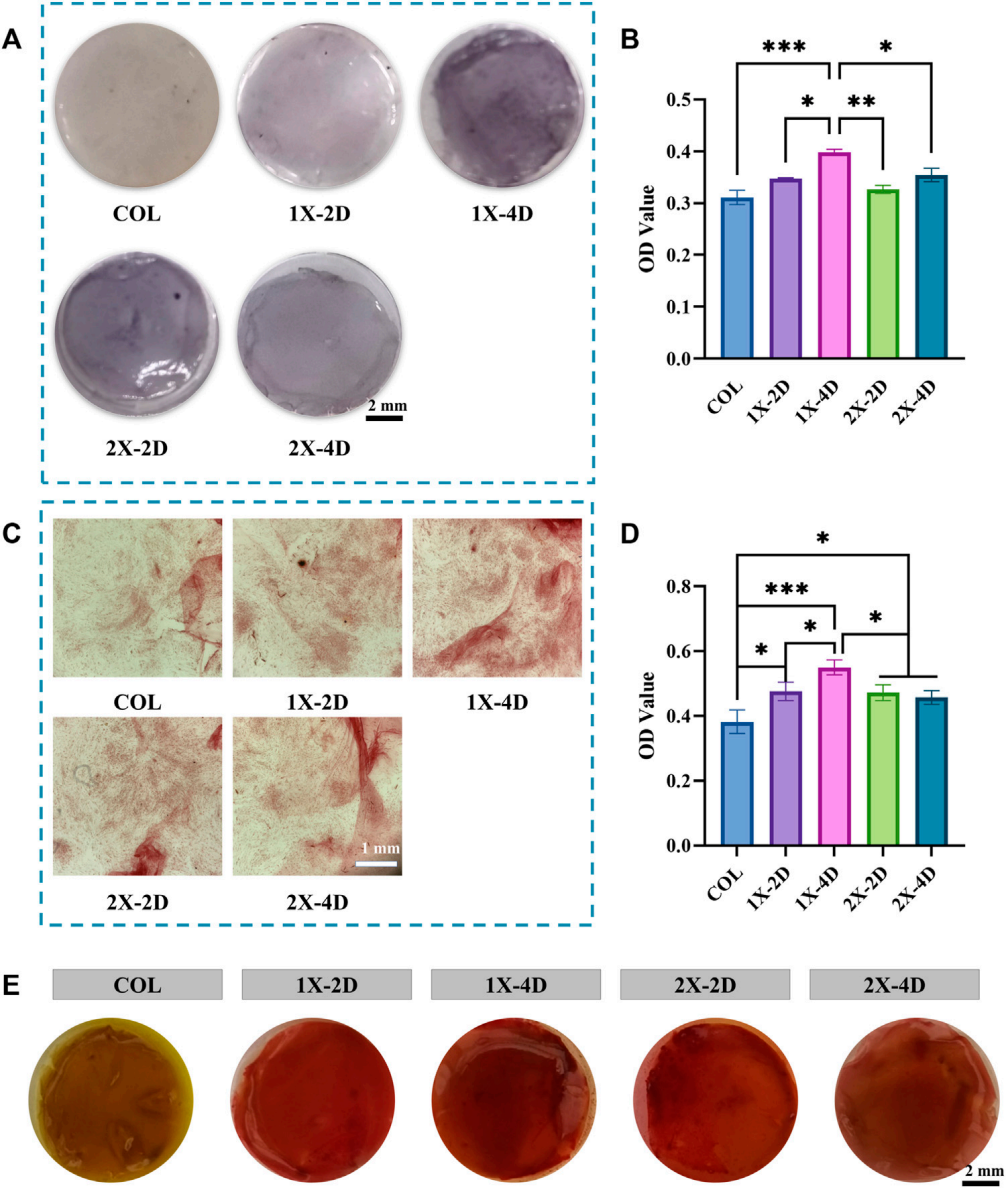
Evaluation of the osteogenic differentiation of BMSCs seeded on COL and I-EMC. **(A)** Alkaline phosphatase (ALP) staining and **(B)** ALP activity determination at 7 days post osteogenic differentiation induction. **(C)** Alizarin red staining and **(D)** quantification of alizarin red in BMSCs cultured with various samples in osteogenic media for 2 weeks. **(E)** Images of different groups post alizarin red S staining; (*p < 0.05, **p < 0.01, ***p < 0.001).

Osteogenic differentiation plays a pivotal role in maintaining the equilibrium of the bone microenvironment. An ideal bone graft material should possess excellent biocompatibility and exhibit outstanding activity during the process of bone formation. Alkaline phosphatase (ALP) is a crucial protein involved in the differentiation of osteoblasts, playing a key role in early bone formation. Figure 7A displays the ALP staining results, where the COL surface shows fewer and lighter blue-purple areas, whereas the I-EMC surface exhibits increased and deeper ALP-positive regions, indicating a more significant ALP expression, especially in the 1X-4D and 2X-2D groups. Semi-quantitative analysis of ALP activity (Figure 7B) indicates that, compared to COL, the I-EMC material significantly enhances ALP activity, particularly in the 1X-4D group, with significant differences (*p* < 0.05). Therefore, compared to COL, I-EMC promotes the early differentiation of BMSCs toward osteogenesis. The 1X-4D sample exhibits remarkable osteogenic induction, with both ALP secretion and activity surpassing those of other groups, indicating the most favorable osteogenic effect, consistent with other study findings (Wang et al., 2019).

Calcium deposition serves as an indicator of late-stage osteogenic differentiation. To evaluate the osteogenic differentiation of BMSCs, we performed alizarin red staining. BMSCs were co-cultured with samples from each group, and after 14 days of osteogenic induction, the underlying cells were stained, as depicted in Figure 7C. The semi-quantitative results are in Figure 7D, revealing that BMSCs co-cultured with I-EMC had richer and denser calcium nodules. Notably, the 1X-4D sample showed the most pronounced calcium deposition. Figure 7E displays the staining images of these nodules. When compared to the COL samples, I-EMC exhibited significantly deeper red-stained areas, with the 1X-4D sample being especially vivid in staining depth and coverage. This evidence underscores the vital role of I-EMC in osteogenic differentiation, with the 1X-4D and 2X-2D samples emerging as the most illustrative.

**FIGURE 8:**
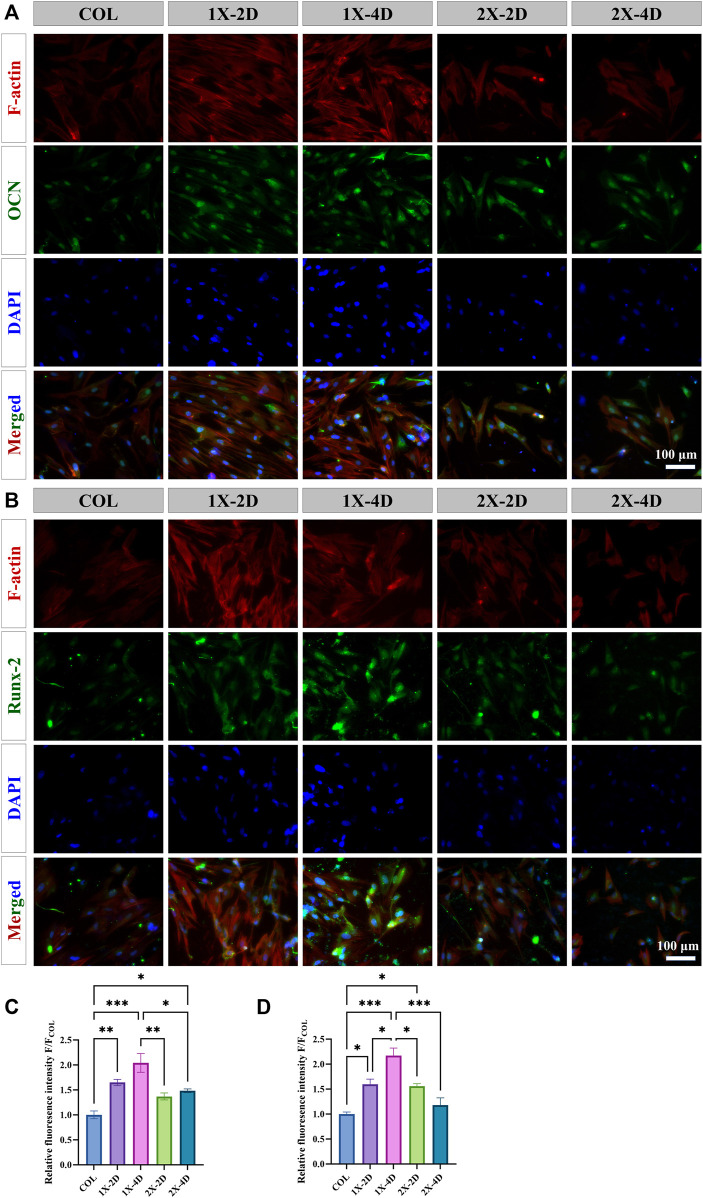
Immunohistochemical staining and quantitative analysis of osteogenic-related proteins in BMSCs cultured in different groups for 14 days, specifically focusing on **(A, C)** OCN and **(B, D)** RUNX-2. (*p < 0.05, **p < 0.01, ***p < 0.001).

During osteogenic differentiation, osteogenic-related proteins play a crucial role. We employed immunofluorescence staining to explore the effect of I-EMC on the osteogenic differentiation of BMSCs, specifically targeting osteocalcin (OCN) and runt-related transcription factor 2 (RUNX-2), both closely linked to bone formation. After 14 days of osteogenic induction, all BMSC groups exhibited OCN expression (Figure 8A) and RUNX-2 expression (Figure 8B). BMSCs cultured on the I-EMC surface showed significantly higher average fluorescence intensities of OCN and RUNX-2 compared to the COL group, particularly in the 1X-2D and 1X-4D samples with the most prominent fluorescence signals (*p* < 0.05). Quantitative analysis indicated that, under 1X-2D, 1X-4D, and 2X-4D culture conditions, BMSCs demonstrated significant increases in OCN expression by 1.64-fold, 2.04-fold, and 1.48-fold, respectively (Figure 8C). Additionally, under 1X-2D, 1X-4D, and 2X-2D culture conditions, the expression of RUNX-2 in BMSCs was also significantly upregulated by 1.59-fold, 2.17-fold, and 1.56-fold, respectively (Figure 8D) (*p* < 0.05), compared to the COL group.

Based on our comprehensive experimental results, all I-EMC samples significantly enhanced the ALP activity of BMSCs, promoted mineral deposition, and increased osteogenic-related protein expression compared to COL, showcasing excellent osteogenic performance. This is consistent with previous research findings. Compared to non-mineralized collagen, the addition of calcium phosphate in collagen significantly enhances the prolonged metabolic activity of BMSCs and amplifies the expression of osteogenesis-associated genes (Weisgerber et al., 2015; de Melo Pereira et al., 2020). *In vivo* studies also support the idea that the combination of HA with collagen more effectively promotes new bone formation (Liu et al., 2016b; de Melo Pereira et al., 2020). This effect is likely due to the inherent osteogenic potential of HA, providing both osteoconductivity and stimulating the expression of osteogenic-related factors (Chai et al., 2011; Huanhuan et al., 2013). Additionally, the characteristics of I-EMC, including the slow release of calcium and phosphate ions, directly influence the cell’s osteogenic differentiation process (Pamela et al., 2010; Chai et al., 2011; Huanhuan et al., 2013; Danoux et al., 2015). These findings further underscore the potential and promising applications of I-EMC in bone tissue engineering and bone graft materials research.

The physicochemical properties of materials play a critical role in determining their biological characteristics. In mineralized collagen, the phase of the mineral substance and its manner of integration with collagen fibers influence the response of BMSCs (Liu et al., 2016c; Santhakumar et al., 2021). Compared to EMC, both hierarchical IMC and IMC exhibit a higher Young’s modulus. Furthermore, they significantly enhance the adhesion, proliferation, and differentiation capabilities of osteogenic-related cells, promoting the expression of osteogenic-associated genes, new bone formation, and the repair of bone fractures (Liu et al., 2013; Liu et al., 2014; Wang Y.-F. et al., 2016; Jin et al., 2019). I-EMC, prepared under various conditions, exhibits distinct microstructures and material compositions. Surface morphology and roughness, influenced by hydroxyapatite, significantly impact cell responses and new bone formation (Liu et al., 2014; Roman et al., 2014; Costa-Rodrigues et al., 2016). Notably, the 1X-2D and 1X-4D samples show significantly higher ALP activity and mineral generation compared to the 2X-2D and 2X-4D samples. This difference can be attributed to the relatively smooth and uniform surface structure of the 1X-2D and 1X-4D samples, along with the presence of nano-sized apatites (Fukui et al., 2008). Additionally, the deposition of larger calcium phosphate particles on the surfaces of 2X-2D and 2X-4D samples significantly increased the surface roughness. Previous *in vivo* studies have suggested that a rough surface facilitates the polarization of monocytes toward M1, while a relatively smooth surface promotes M2 polarization (Sun et al., 2016; Jin et al., 2019). M1 polarization may release certain cytokines inhibiting the osteogenic process, whereas M2 polarization secretes cytokines that promote osteogenic differentiation, fostering bone formation. Furthermore, studies suggest that the composite material exhibits optimal osteogenic differentiation and bone tissue regeneration effects only when the collagen-to-hydroxyapatite ratio is appropriate (Roman et al., 2014; Wang Y. et al., 2016; Ma et al., 2018). Based on thermal gravimetric analysis, we found that the 2X-4D samples have higher mineral content, which might not be the optimal mineral-to-collagen ratio. Similar trends were observed in the expressions of OCN and RUNX-2.

Furthermore, when comparing the 1X-2D and 1X-4D samples, we found that the 1X-4D sample exhibited significantly enhanced osteogenic performance, especially in terms of ALP activity and RUNX-2 expression (*p* < 0.05). We posit that the enhanced osteogenic performance of 1X-4D is likely tied to its optimal mineral-to-collagen ratio. Drawing upon our past investigations, especially insights from EDS mapping images, we identified that 1X-4D displays a more uniform distribution of hydroxyapatite crystals compared to 1X-2D. Such uniformity likely plays a significant role in the enhanced osteogenic efficacy of 1X-4D. This indicates that extending the mineralization time during the preparation of I-EMC with 1× mineralization solution can enhance its osteogenic potential. However, in comparison, the 2X-4D did not show stronger osteogenic activity than 2X-2D. This is likely due to the rough surface structure and uneven mineralization of I-EMC prepared with the 2× mineralization solution. Additionally, the presence of a large amount of mineral outside the fibers and a decrease in the stabilizing effect of PAA may prevent a significant increase in nano-sized apatite within the fibers, even with extended mineralization time. These findings suggest that the mineralization time and concentration of mineralization solution play a crucial role in optimizing the osteogenic performance of I-EMC materials.

Mineralized collagen is a high-quality material for bone repair, with numerous products available on the market. Some have received regulatory approval and are in commercial production. In clinical practice, especially in the fields of orthopedics, dentistry, and neurosurgery, artificial bone has achieved significant success (Qiu et al., 2015). Mineralized collagen has demonstrated reliable effectiveness and fewer complications in clinical applications such as open reduction and internal fixation of bone fractures, grafting after benign bone tumor resection, total hip arthroplasty, and internal fixation fusion procedures (Huang et al., 2015; Ghate and Cui, 2017; Pan et al., 2018; Gao et al., 2020). However, current mineralized collagen protein products are primarily prepared using traditional methods, such as collagen mixture or co-precipitation with hydroxyapatite (Niu et al., 2023), emphasizing compositional biomimicry. In contrast, I-EMC, with both compositional and structural biomimicry, demonstrates superior mechanical performance and biological activity. Nonetheless, there is still a need for further exploration and research in clinical applications. This study found that increasing the concentration of the mineralization solution can shorten the mineralization time and achieve intrafiber mineralization, facilitating the commercial production and clinical application of mineralized collagen products with dual biomimicry. Additionally, the research revealed that the osteogenic performance of mineralized collagen is not only related to its degree of mineralization but also closely associated with its surface nanostructure. These findings provide crucial insights for advancing the development of mineralized collagen products with enhanced biomimetic and osteogenic properties.

## 4 Conclusion

In this research, the primary objective was to examine how specific parameters during collagen mineralization influence the material’s microstructure, its physicochemical properties, and the creation of an essential osteogenic microenvironment. The role of microstructure in cell behavior was explored, aiming to define the “condition-structure-performance” relationship of I-EMC. The findings highlighted the optimal mineralization conditions for achieving the best bone-forming performance. While the study offers valuable insights, it also has limitations. Specifically, a bone repair model was not used when assessing the osteogenic potential of I-EMC, an area we aim to address in our future research. These findings will drive the commercial production and clinical application of mineralized collagen products with dual biomimicry. This lays the foundation for the development of the next-generation of bone substitute materials based on mineralized collagen protein. It provides robust support for the advancement of bone tissue engineering and transplant materials, highlighting a promising future in the treatment of various extensive bone defects.

## Data Availability

The original contributions presented in the study are included in the article/Supplementary Material, further inquiries can be directed to the corresponding author.

## References

[B1] Agarwal R. García A. J. (2015). Biomaterial strategies for engineering implants for enhanced osseointegration and bone repair. Adv. Drug Deliv. Rev. 94, 53–62. 10.1016/j.addr.2015.03.013 25861724 PMC4598264

[B2] Cerutti C. Ridley A. J. (2017). Endothelial cell-cell adhesion and signaling. Exp. Cell Res. 358, 31–38. 10.1016/j.yexcr.2017.06.003 28602626 PMC5700119

[B3] Chai Y. C. Roberts S. J. Schrooten J. Luyten F. P. (2011). Probing the osteoinductive effect of calcium phosphate by using an *in vitro* biomimetic model. Tissue Eng. Part A 17, 1083–1097. 10.1089/ten.tea.2010.0160 21091326

[B4] Chen L. Zeng Z. Li W. (2023). Poly(acrylic acid)-assisted intrafibrillar mineralization of type I collagen: a review. Macromol. Rapid Commun. 44, e2200827. 10.1002/marc.202200827 36662644

[B5] Chen Z. Luo Q. Lin C. Kuang D. Song G. (2016). Simulated microgravity inhibits osteogenic differentiation of mesenchymal stem cells via depolymerizing F-actin to impede TAZ nuclear translocation. Sci. Rep. 6, 30322. 10.1038/srep30322 27444891 PMC4957213

[B6] Costa-Rodrigues J. Carmo S. Perpétuo I. P. Monteiro F. J. Fernandes M. H. (2016). Osteoclastogenic differentiation of human precursor cells over micro- and nanostructured hydroxyapatite topography. Biochimica Biophysica Acta 1860, 825–835. 10.1016/j.bbagen.2016.01.014 26801877

[B7] Danoux C. B. S. S. Bassett D. C. Othman Z. Rodrigues A. I. Reis R. L. Barralet J. E. (2015). Elucidating the individual effects of calcium and phosphate ions on hMSCs by using composite materials. Acta Biomater. 17, 1–15. 10.1016/j.actbio.2015.02.003 25676583

[B8] de Melo Pereira D. Eischen-Loges M. Birgani Z. T. Habibovic P. (2020). Proliferation and osteogenic differentiation of hMSCs on biomineralized collagen. Front. Bioeng. Biotechnol. 8, 554565. 10.3389/fbioe.2020.554565 33195119 PMC7644787

[B9] Dhand C. Ong S. T. Dwivedi N. Diaz S. M. Venugopal J. R. Navaneethan B. (2016). Bio-inspired *in situ* crosslinking and mineralization of electrospun collagen scaffolds for bone tissue engineering. Biomaterials 104, 323–338. 10.1016/j.biomaterials.2016.07.007 27475728

[B10] dos Remedios C. G. Chhabra D. Kekic M. Dedova I. V. Tsubakihara M. Berry D. A. (2003). Actin binding proteins: regulation of cytoskeletal microfilaments. Physiol. Rev. 83, 433–473. 10.1152/physrev.00026.2002 12663865

[B11] Du T. Niu X. Li Z. Li P. Feng Q. Fan Y. (2018). Crosslinking induces high mineralization of apatite minerals on collagen fibers. Int. J. Biol. Macromol. 113, 450–457. 10.1016/j.ijbiomac.2018.02.136 29481955

[B12] Du T. Niu Y. Liu Y. Yang H. Qiao A. Niu X. (2022). Physical and chemical characterization of biomineralized collagen with different microstructures. J. Funct. Biomater. 13, 57. 10.3390/jfb13020057 35645265 PMC9149879

[B13] Fukui N. Sato T. Kuboki Y. Aoki H. (2008). Bone tissue reaction of nano-hydroxyapatite/collagen composite at the early stage of implantation. Bio-medical Mater. Eng. 18, 25–33. 18198404

[B14] Gao C. Qiu Z. Y. Hou J. W. Tian W. Kou J. M. Wang X. (2020). Clinical observation of mineralized collagen bone grafting after curettage of benign bone tumors. Regen. Biomater. 7, 567–575. 10.1093/rb/rbaa031 33365142 PMC7748453

[B15] Ghate N. S. Cui H. (2017). Mineralized collagen artificial bone repair material products used for fusing the podarthral joints with internal fixation-a case report. Regen. Biomater. 4, 295–298. 10.1093/rb/rbx015 29026643 PMC5633689

[B16] Höhling H. J. Barckhaus R. H. Krefting E. R. Althoff J. Quint P. (1990). Collagen mineralization: aspects of the structural relationship between collagen and the apatitic crystallites. Springer US.

[B17] Hu C. Zilm M. Wei M. (2016). Fabrication of intrafibrillar and extrafibrillar mineralized collagen/apatite scaffolds with a hierarchical structure. J. Biomed. Mater. Res. Part A 104, 1153–1161. 10.1002/jbm.a.35649 26748775

[B18] Huang C. Qin L. Yan W. Weng X. Huang X. (2015). Clinical evaluation following the use of mineralized collagen graft for bone defects in revision total hip arthroplasty. Regen. Biomater. 2, 245–249. 10.1093/rb/rbv022 26816647 PMC4676328

[B19] Huang R. L. Kobayashi E. Liu K. Li Q. (2016). Bone graft prefabrication following the *in vivo* bioreactor principle. EBioMedicine 12, 43–54. 10.1016/j.ebiom.2016.09.016 27693103 PMC5078640

[B20] Huanhuan L. Peng H. Wu Y. Zhang C. Cai Y. Xu G. (2013). The promotion of bone regeneration by nanofibrous hydroxyapatite/chitosan scaffolds by effects on integrin-BMP/Smad signaling pathway in BMSCs. Biomaterials 34, 4404–4417. 10.1016/j.biomaterials.2013.02.048 23515177

[B21] Jee S.-S. Thula T. T. Gower L. B. (2010). Development of bone-like composites via the polymer-induced liquid-precursor (PILP) process. Part 1: influence of polymer molecular weight. Acta Biomater. 6, 3676–3686. 10.1016/j.actbio.2010.03.036 20359554

[B22] Jin S.-S. He D.-Q. Luo D. Wang Y. Yu M. Guan B. (2019). A biomimetic hierarchical nanointerface orchestrates macrophage polarization and mesenchymal stem cell recruitment to promote endogenous bone regeneration. Acs Nano 13, 6581–6595. 10.1021/acsnano.9b00489 31125522

[B23] Kikuchi M. Matsumoto H. N. Yamada T. Koyama Y. Takakuda K. Tanaka J. (2004). Glutaraldehyde cross-linked hydroxyapatite/collagen self-organized nanocomposites. Biomaterials 25, 63–69. 10.1016/s0142-9612(03)00472-1 14580909

[B24] Kim J. W. Han Y. S. Lee H. M. Kim J. K. Kim Y. J. (2021). Effect of morphological characteristics and biomineralization of 3D-printed gelatin/hyaluronic acid/hydroxyapatite composite scaffolds on bone tissue regeneration. Int. J. Mol. Sci. 22, 6794. 10.3390/ijms22136794 34202759 PMC8267715

[B25] Li D. Zhang K. Shi C. Liu L. Yan G. Liu C. (2018). Small molecules modified biomimetic gelatin/hydroxyapatite nanofibers constructing an ideal osteogenic microenvironment with significantly enhanced cranial bone formation. Int. J. Nanomedicine 13, 7167–7181. 10.2147/ijn.s174553 30464466 PMC6228053

[B26] Li Y. Thula T. T. Jee S. Perkins S. L. Aparicio C. Douglas E. P. (2012). Biomimetic mineralization of woven bone-like nanocomposites: role of collagen cross-links. Biomacromolecules 13, 49–59. 10.1021/bm201070g 22133238

[B27] Liu S. Sun Y. Fu Y. Chang D. Fu C. Wang G. (2016a). Bioinspired collagen-apatite nanocomposites for bone regeneration. J. Endod. 42, 1226–1232. 10.1016/j.joen.2016.04.027 27377439

[B28] Liu Y. Kim Y.-K. Dai L. Li N. Khan S. O. Pashley D. H. (2011). Hierarchical and non-hierarchical mineralisation of collagen. Biomaterials 32, 1291–1300. 10.1016/j.biomaterials.2010.10.018 21040969 PMC3003335

[B29] Liu Y. Liu S. Luo D. Xue Z. Yang X. Cu L. (2016b). Hierarchically staggered nanostructure of mineralized collagen as a bone-grafting scaffold. Adv. Mater. 28, 8740–8748. 10.1002/adma.201602628 27530607

[B30] Liu Y. Luo D. Kou X.-X. Wang X.-D. Tay F. R. Sha Y.-L. (2013). Hierarchical intrafibrillar nanocarbonated apatite assembly improves the nanomechanics and cytocompatibility of mineralized collagen. Adv. Funct. Mater. 23, 1404–1411. 10.1002/adfm.201201611

[B31] Liu Y. Luo D. Liu S. Fu Y. Kou X. Wang X. (2014). Effect of nanostructure of mineralized collagen scaffolds on their physical properties and osteogenic potential. J. Biomed. Nanotechnol. 10, 1049–1060. 10.1166/jbn.2014.1794 24749399

[B32] Liu Y. Luo D. Wang T. (2016c). Hierarchical structures of bone and bioinspired bone tissue engineering. Small (Weinheim der Bergstrasse, Ger. 12, 4611–4632. 10.1002/smll.201600626 27322951

[B33] Ma L. Wang X. Zhao N. Zhu Y. Qiu Z. Li Q. (2018). Integrating 3D printing and biomimetic mineralization for personalized enhanced osteogenesis, angiogenesis, and osteointegration. Acs Appl. Mater. Interfaces 10, 42146–42154. 10.1021/acsami.8b17495 30507136 PMC6456406

[B34] Niu Y. Du T. Liu Y. (2023). Biomechanical characteristics and analysis approaches of bone and bone substitute materials. J. Funct. Biomater. 14, 212. 10.3390/jfb14040212 37103302 PMC10146666

[B35] Olszta M. J. Douglas E. P. Gower L. B. (2003). Scanning electron microscopic analysis of the mineralization of type I collagen via a polymer-induced liquid-precursor (PILP) process. Calcif. Tissue Int. 72, 583–591. 10.1007/s00223-002-1032-7 12616327

[B36] Oosterlaken B. M. Vena M. P. de With G. (2021). *In vitro* mineralization of collagen. Adv. Mater. 33, e2004418. 10.1002/adma.202004418 33711177 PMC11469168

[B37] Pamela H. David C. Charles J. B. Doillon C. McKee M. D. Barralet J. E. (2010). Collagen biomineralization *in vivo* by sustained release of inorganic phosphate ions. Adv. Mater. 22, 1858–1862. 10.1002/adma.200902778 20512962

[B38] Pan Y. X. Yang G. G. Li Z. W. Shi Z. M. Sun Z. D. (2018). Clinical observation of biomimetic mineralized collagen artificial bone putty for bone reconstruction of calcaneus fracture. Regen. Biomater. 5, 61–67. 10.1093/rb/rbx033 29644087 PMC5888141

[B39] Perrier A. Dumas V. Linossier M. T. Fournier C. Jurdic P. Rattner A. (2010). Apatite content of collagen materials dose-dependently increases pre-osteoblastic cell deposition of a cement line-like matrix. Bone 47, 23–33. 10.1016/j.bone.2010.03.010 20303420

[B40] Pollard T. D. Cooper J. A. (2009). Actin, a central player in cell shape and movement. Science 326, 1208–1212. 10.1126/science.1175862 19965462 PMC3677050

[B41] Qi Y. Ye Z. Fok A. Holmes B. N. Espanol M. Ginebra M. P. (2018). Effects of molecular weight and concentration of poly(acrylic acid) on biomimetic mineralization of collagen. ACS Biomater. Sci. Eng. 4, 2758–2766. 10.1021/acsbiomaterials.8b00512 30581990 PMC6298758

[B42] Qiu Z.-Y. Cui Y. Tao C.-S. Zhang Z.-Q. Tang P.-F. Mao K.-Y. (2015). Mineralized collagen: rationale, current status, and clinical applications. Mater. (Basel, Switz. 8, 4733–4750. 10.3390/ma8084733 PMC5455477 28793468

[B43] Reznikov N. Shahar R. Weiner S. (2014a). Bone hierarchical structure in three dimensions. Acta Biomater. 10, 3815–3826. 10.1016/j.actbio.2014.05.024 24914825

[B44] Reznikov N. Shahar R. Weiner S. (2014b). Three-dimensional structure of human lamellar bone: the presence of two different materials and new insights into the hierarchical organization. Bone 59, 93–104. 10.1016/j.bone.2013.10.023 24211799

[B45] Roman S. M. Surmeneva M. A. Ivanova A. A. (2014). Significance of calcium phosphate coatings for the enhancement of new bone osteogenesis--a review. Acta Biomater. 10, 557–579. 10.1016/j.actbio.2013.10.036 24211734

[B46] Santhakumar S. Oyane A. Nakamura M. Yoshino Y. Alruwaili M. K. Miyaji H. (2021). Bone tissue regeneration by collagen scaffolds with different calcium phosphate coatings: amorphous calcium phosphate and low-crystalline apatite. Materials 14, 5860. 10.3390/ma14195860 34640257 PMC8510234

[B47] Sun Y. Liu S. Fu Y. Kou X.-X. He D.-Q. Wang G.-N. (2016). Mineralized collagen regulates macrophage polarization during bone regeneration. J. Biomed. Nanotechnol. 12, 2029–2040. 10.1166/jbn.2016.2296 29364617

[B48] Thrivikraman G. Athirasala A. Gordon R. Zhang L. Bergan R. Keene D. R. (2019). Rapid fabrication of vascularized and innervated cell-laden bone models with biomimetic intrafibrillar collagen mineralization. Nat. Commun. 10, 3520. 10.1038/s41467-019-11455-8 31388010 PMC6684598

[B49] Wang J. Qu Y. Chen C. Sun J. Pan H. Shao C. (2019). Fabrication of collagen membranes with different intrafibrillar mineralization degree as a potential use for GBR. Mater. Sci. Eng. C, Mater. Biol. Appl. 104, 109959. 10.1016/j.msec.2019.109959 31500040

[B50] Wang Y. Van Manh N. Wang H. Zhong X. Zhang X. Li C. (2016b). Synergistic intrafibrillar/extrafibrillar mineralization of collagen scaffolds based on a biomimetic strategy to promote the regeneration of bone defects. Int. J. nanomedicine 11, 2053–2067. 10.2147/ijn.s102844 27274235 PMC4869647

[B51] Wang Y.-F. Wang C.-Y. Wan P. Wang S.-G. Wang X.-M. (2016a). Comparison of bone regeneration in alveolar bone of dogs on mineralized collagen grafts with two composition ratios of nano-hydroxyapatite and collagen. Regen. Biomater. 3, 33–40. 10.1093/rb/rbv025 26816654 PMC4723277

[B52] Weisgerber D. W. Caliari S. R. Harley B. A. C. (2015). Mineralized collagen scaffolds induce hMSC osteogenesis and matrix remodeling. Biomaterials Sci. 3, 533–542. 10.1039/c4bm00397g PMC4412464 25937924

[B53] Wu J. Chen T. Wang Z. Chen X. Qu S. Weng J. (2020). Joint construction of micro-vibration stimulation and BCP scaffolds for enhanced bioactivity and self-adaptability tissue engineered bone grafts. J. Mater Chem. B 8, 4278–4288. 10.1039/d0tb00223b 32309841

[B54] Xia Z. Yu X. Jiang X. Brody H. D. Rowe D. W. Wei M. (2013). Fabrication and characterization of biomimetic collagen-apatite scaffolds with tunable structures for bone tissue engineering. Acta Biomater. 9, 7308–7319. 10.1016/j.actbio.2013.03.038 23567944 PMC3738228

[B55] Xiao Q. Zhang Y. Qi X. Chen Y. Sheng R. Xu R. (2020). AFF4 regulates osteogenic differentiation of human dental follicle cells. Int. J. Oral Sci. 12, 20. 10.1038/s41368-020-0083-9 32606293 PMC7327054

[B56] Xu S.-J. Qiu Z.-Y. Wu J.-J. Kong X.-D. Weng X.-S. Cui F.-Z. (2016). Osteogenic differentiation gene expression profiling of hMSCs on hydroxyapatite and mineralized collagen. Tissue Eng. Part A 22, 170–181. 10.1089/ten.tea.2015.0237 26529501

[B57] Xuan Y. Li L. Ma M. Cao J. Zhang Z. (2021). Hierarchical intrafibrillarly mineralized collagen membrane promotes guided bone regeneration and regulates M2 macrophage polarization. Front. Bioeng. Biotechnol. 9, 781268. 10.3389/fbioe.2021.781268 35155400 PMC8826568

[B58] Ye B. Luo X. Li Z. Zhuang C. Li L. Lu L. (2016). Rapid biomimetic mineralization of collagen fibrils and combining with human umbilical cord mesenchymal stem cells for bone defects healing. Mater. Sci. Eng. C-Materials Biol. Appl. 68, 43–51. 10.1016/j.msec.2016.05.104 27523994

[B59] Ye Z. Zhu X. Mutreja I. Boda S. K. Fischer N. G. Zhang A. (2021). Biomimetic mineralized hybrid scaffolds with antimicrobial peptides. Bioact. Mater. 6, 2250–2260. 10.1016/j.bioactmat.2020.12.029 33553813 PMC7829078

[B60] Yu L. Rowe D. W. Perera I. P. Zhang J. Suib S. L. Xin X. (2020). Intrafibrillar mineralized collagen-hydroxyapatite-based scaffolds for bone regeneration. Acs Appl. Mater. Interfaces 12, 18235–18249. 10.1021/acsami.0c00275 32212615

[B61] Yu L. Wei M. (2021). Biomineralization of collagen-based materials for hard tissue repair. Int. J. Mol. Sci. 22, 944. 10.3390/ijms22020944 33477897 PMC7833386

[B62] Zandi N. Sani E. S. Mostafavi E. Ibrahim D. M. Saleh B. Shokrgozar M. A. (2021). Nanoengineered shear-thinning and bioprintable hydrogel as a versatile platform for biomedical applications. Biomaterials 267, 120476. 10.1016/j.biomaterials.2020.120476 33137603 PMC7846391

[B63] Zhang Z. Li Z. Zhang C. Liu J. Bai Y. Li S. (2018). Biomimetic intrafibrillar mineralized collagen promotes bone regeneration via activation of the Wnt signaling pathway. Int. J. nanomedicine 13, 7503–7516. 10.2147/ijn.s172164 30538446 PMC6257138

[B64] Zhang Z. Zhang S. Li Z. Li S. Liu J. Zhang C. (2019). Osseointegration effect of biomimetic intrafibrillarly mineralized collagen applied simultaneously with titanium implant: a pilot *in vivo* study. Clin. Oral Implants Res. 30, 637–648. 10.1111/clr.13449 31034662

[B65] Zhao Y. Li Z. Jiang Y. Liu H. Feng Y. Wang Z. (2020). Bioinspired mineral hydrogels as nanocomposite scaffolds for the promotion of osteogenic marker expression and the induction of bone regeneration in osteoporosis. Acta Biomater. 113, 614–626. 10.1016/j.actbio.2020.06.024 32565370

[B66] Zhou Q. Ren X. Bischoff D. Weisgerber D. W. Yamaguchi D. T. Miller T. A. (2017). Nonmineralized and mineralized collagen scaffolds induce differential osteogenic signaling pathways in human mesenchymal stem cells. Adv. Healthc. Mater. 6. 10.1002/adhm.201700641 PMC5831257 28945007

[B67] Zhu X. Wang C. Bai H. Zhang J. Wang Z. Li Z. (2023). Functionalization of biomimetic mineralized collagen for bone tissue engineering. Mater. Today Bio 20, 100660. 10.1016/j.mtbio.2023.100660 PMC10199226 37214545

